# An Update of Anthraquinone Derivatives Emodin, Diacerein, and Catenarin in Diabetes

**DOI:** 10.1155/2021/3313419

**Published:** 2021-09-20

**Authors:** Miquel Martorell, Natalia Castro, Montserrat Victoriano, Xavier Capó, Silvia Tejada, Sara Vitalini, Raffaele Pezzani, Antoni Sureda

**Affiliations:** ^1^Department of Nutrition and Dietetics, Faculty of Pharmacy, University of Concepción, Concepción 4070386, Chile; ^2^Centre for Healthy Living, University of Concepción, Concepción 4070386, Chile; ^3^Research Group in Community Nutrition and Oxidative Stress, University Research Institute of Health Sciences (IUNICS), University of the Balearic Islands, Palma de Mallorca 07122, Spain; ^4^Laboratory of Neurophysiology, Department of Biology, University Research Institute of Health Sciences (IUNICS), University of Balearic Islands, Palma E-07122, Balearic Islands, Spain; ^5^CIBEROBN (Physiopathology of Obesity and Nutrition), Instituto de Salud Carlos III, Madrid E-28029, Spain; ^6^Research Institute of the Balearic Islands, Palma de Mallorca E-07120, Spain; ^7^Department of Agricultural and Environmental Sciences, Università degli Studi di Milano, Via G. Celoria 2 20133, Milan, Italy; ^8^Phytotherapy Lab (PhT-Lab), Endocrinology Unit, Department of Medicine (DIMED), University of Padova, via Ospedale 105, Padova 35128, Italy; ^9^AIROB, Associazione Italiana per la Ricerca Oncologica di Base, Padova, Italy

## Abstract

Diabetes is part of metabolic diseases and is characterized by high blood sugar levels over a prolonged period as result of an insulin-deficient production or an inappropriate response to insulin by our cells. This chronic disease was the direct cause of 1.6 million deaths in 2016 as reported by the World Health Organization. Emodin is a natural product and active ingredient of various Chinese herbs with the chemical formula 1,3,8-trihydroxy-6-methylanthraquinone. Diacerein is another naturally occurring anthraquinone (1,8-diacetoxy-3-carboxyanthraquinone) commonly used as commercial drug to treat osteoarthritis. These two anthraquinone derivatives have been shown to exert antidiabetic activities. Emodin seems to enhance the glucose tolerance and insulin sensibility via activation of PPAR*γ* and modulation of metabolic-related genes. Diacerein seems to decrease inflammatory cytokines and increase insulin secretion enhancing insulin sensibility and therefore improving glucose control. Other naturally occurring anthraquinone derivatives, such as catenarin (1,4,6,8-tetrahydroxy-3-methylanthraquinone), have been shown to have antidiabetic activities although few studies have been performed. The synthesis of new emodin derivatives is increasing, but these new molecules have not been tested for diabetes treatment. In the current work, available literature on anthraquinone derivatives' effects in diabetes disease is reviewed. Moreover, we discuss the chemistry, food sources, bioavailability, and toxicity of the naturally occurring anthraquinone with antidiabetic effects.

## 1. Introduction

The prevalence of diabetes has increased rapidly and it is expected to double within the next 20 years due to the increased risk of age and obesity with significant rises in cardiovascular disease [1]. This condition is produced by a relative deficiency of insulin. This means an impaired insulin secretion through a dysfunction of the pancreatic *β*-cell and an impaired insulin action through insulin resistance [2]. Oxidative stress has long been considered an important factor driving obesity-related insulin resistance and subsequent diabetes. It is well known that obesity causes features of metabolic dysfunction in the adipose tissue. These include reduced insulin-stimulated glucose transport and expression of glucose transporter type 4 (GLUT4), altered expression of adipokines, and adipocyte hypertrophy [3]. Several studies have confirmed antidiabetic actions of natural products with recognized anti-inflammatory activity [4].

Oxidative stress associated with diabetes is the consequence of some abnormalities such as inflammation, insulin resistance, dyslipidemia, and hyperglycemia [5]. It has been demonstrated that high glucose levels caused an anion superoxide overproduction by the mitochondrial electron chain that affects many metabolic and signaling pathways involved in diabetic complications [5]. The main key factors implicated in the increase of reactive oxygen species (ROS) production are mitochondrial respiratory chain, NAD(P)H oxidases, defects in polyol pathway, and advanced glycation end-products alterations on this polyol pathway. In addition, deficiencies in the function of NAD(P)H oxidases are related to a decrease in the glutathione reductase and glutathione peroxidase system, altering the reduced/oxidized glutathione ratio. Altogether, they can increase ROS production [5]. This increase in the ROS levels could be directly associated with the proinflammatory state observed in diabetic patients, frequently due to the decrease in adiponectin levels which have anti-inflammatory properties [6]. Both ROS and reactive nitrogen species (RNS) could affect various intracellular pathways such as transcription factors (nuclear factor kappa beta (NF-*κ*B), forkhead box O (FOXO), new Ets-related factor (Nerf-2)), mitogen-activated kinases, synthesis of various cytokines, among others [7–9]. High levels of interleukin 6 (IL-6), IL-1*β,* and tumor necrosis factor *α* (TNF-*α*) have been observed in diabetic patients [10]. Many pieces of evidence indicate that high TNF-*α* and IL-6 levels are related to health complications of diabetes, such as renal dysfunction, retinopathy, and cardiovascular disease [11–14]. Furthermore, it has been observed that high TNF-*α* could decrease adiponectin expression and plasma levels [6]. C-reactive protein (CRP) is another inflammatory marker that increases in diabetes; indeed, CRP is considered the best epidemiological marker of type 2 diabetes (T2DM) associated with cardiovascular diseases nowadays [15].

Advanced glycoxidation and lipoxidation end-products (AGEs and ALEs) are a heterogeneous group of compounds also used as biomarkers related to oxidative stress [16] and diabetic glomerular lesions [17].

**Figure 1:**
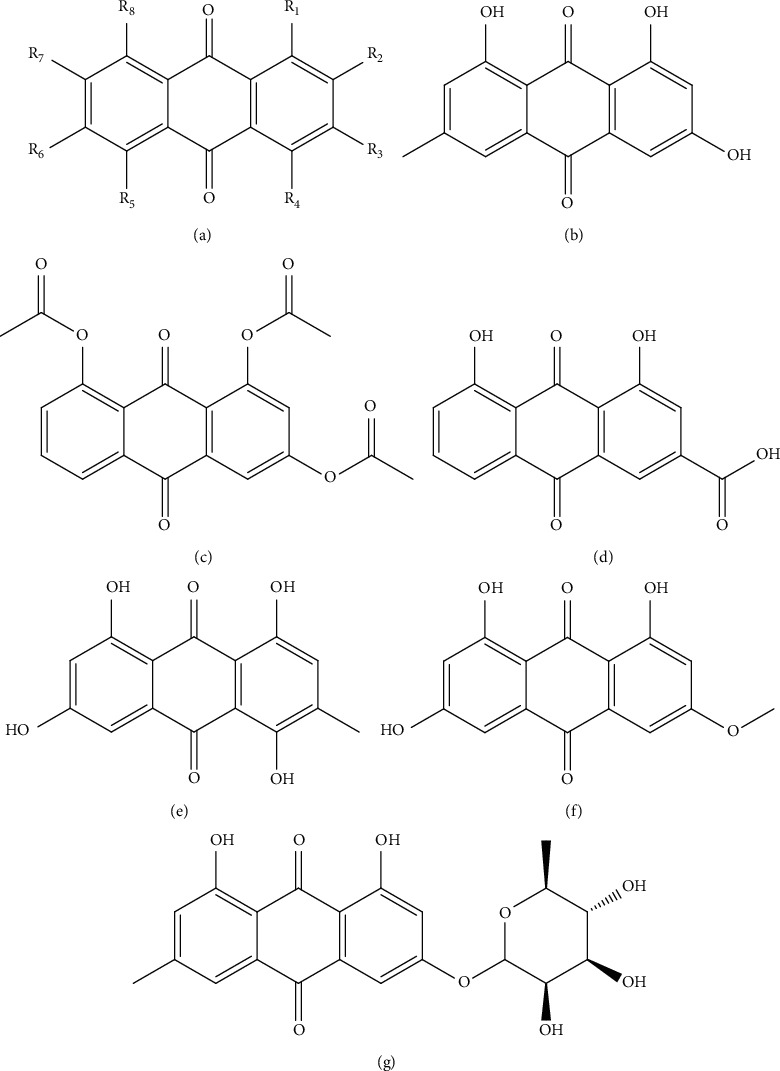
Structures of 9, 10-anthracenedione (a), emodin (b), diacerein (c), rhein (d), catenarin (e), physcion (f), and cascarin (g).

Anthraquinones, also called anthracenediones or dioxoanthracenes, are polycyclic aromatic hydrocarbons that represent a class of the quinone family. The essential structure 9,10-anthracenedione (C_14_H_8_O_2_), also called 9,10-dioxoanthracene, is based on three benzene rings that include two ketone groups on the central ring (Figure 1(a)) [18]. The diversity of the anthraquinone derivatives relies on the nature and the position of the substituents that replace the hydrogen atoms on the basic structure such –OH, –CH_3_, –OCH_3_, –CH_2_OH, –CHO, –COOH, or more complex groups [18]. When *n* hydrogen atoms are replaced by hydroxyl groups, the molecule is called hydroxyanthraquinone and their derivative structures absorb visible [18]. Naturally occurring anthraquinones are a group of secondary metabolites structurally related to the basic structure 9,10-anthracenedione and their glycosides. So far, more than 80 naturally occurring anthraquinones have been identified and isolated from lichens, fungi, or medicinal plants (e.g., *Polygonaceae, Leguminosae, Rhamnaceae*, *Rubiaceae*, *Fabaceae*, and *Xanthorrhoeaceae*) [19–23]. Some of these include emodin, diacerein, catenarin, physcion, cascarin, and rhein (Figure 1).

The biosynthetic pathway of naturally occurring anthraquinones is not fully elucidated yet; different biosynthetic mechanisms are proposed as polyketide or shikimate pathways [19, 24]. Anthraquinones are biosynthesized by the cyclization of linear octa-*β*-ketoacyl CoA intermediates from the addition of one acetyl-CoA to three malonyl-CoA or by the addition of succinyl benzoic acid (C_11_H_10_O_5_), resulting from shikimic acid (C_7_H_10_O_5_) and *α*-ketoglutaric acid (C_5_H_6_O_5_), to mevalonic acid (C_6_H_12_O_4_) [19, 24]. The anthraquinones biosynthesized by the polyketide pathway often exhibit substitutions in both rings A and C [24].

Anthraquinones are being studied since many plants and herbal preparations containing them have been traditionally used for different diseases [25, 26]. Among them, isolated pure emodin or its metabolites are compounds that have been described to possess a free radical scavenging activity [27, 28] and an anti-inflammatory activity [29]. In fact, emodin has been tested to investigate its implication in diabetes. Some works have related the emodin activity to the inhibition of different protein kinases activation [30], which are triggered by environmental factors, such as oxidants and inflammatory processes. Both activities may justify the use of emodin for treating diabetes and diabetic complications, as it has been done traditionally. However, doses and treatment duration should be taken into account since hepatotoxicity induced by emodin in cells, and animal models have been described [31, 32].

## 2. Methods

This review summarized available literature on emodin, diacerein, catenarin and their potential effects on diabetes treatment. Different databases (Cochrane, EMBASE, Google Scholar, Medline, Pubmed, Science Direct, Web of Science) were interrogated with the combined use of different keywords, such as anthraquinone/s, diabetes, antidiabetic, catenarin, clinical trial, diacerein, emodin, glucose, glycaemic control, human study/ies, hypoglycaemic, insulin sensitivity, insulin resistance, mechanism of action.

## 3. Food Sources, Bioavailability, and Toxicity of Anthraquinones

Biologically active anthraquinone derivatives have been identified in bacteria, fungi, and insects. Food sources included extracts of rhubarb, aloe or buckthorn, and other herbal products such as roots, bark, or dried leaves of senna, cascara, frangula [33]. Recently, Greco, Turrini, Catanzaro, and Fimognari [34] reviewed the pharmacological and toxicity of marine-derived anthraquinones. These derivatives are unwell absorbed from the gastrointestinal tract, but they are hewed by the gut bacteria to produce aglycones that are simply absorbed and considered responsible for some therapeutic related properties. Therapeutic properties of anthraquinones include laxative, anticancer, anti-inflammatory, antiarthritic, antifungal, antibacterial, and antiviral [35]. More specifically, laxative properties have been used for many centuries; this effect is caused by two independent mechanisms, an accelerated motility colonic transit and alterations in colonic absorption and secretion. The effect on secretion and absorption is principally induced by a direct interaction between the laxatives and the epithelial cells, while motility changes are caused indirectly by epithelial cell damage that induces watery diarrhea [36]. Absorbed anthraquinones that enter into systemic circulation cause a rapid depletion of extracellular potassium via gastrointestinal routes leading to diarrhea [37]. Apart from these beneficial effects, anthraquinones may produce potential damage to cells because of the close similarity in structure between the toxic analogue, anthracene [38]. The well-known toxicity effects associated with quinone-containing compounds are trouble for safe pharmaceutical use. Toxicity has been related to redox cycling, mutagenic and genotoxic effects; to sum, anthraquinone derivatives can produce a ROS excess, forming complexes with iron that undergo a redox cycling and oxygen radicals generation [39]. Other pathway has been established related to the interaction in the cell differentiation and the interference with DNA unwinding/DNA strand separation and DNA helicase [40]. Shukla, Asthana, Gupta, Dwivedi, Tripathi, and Das [37] reviewed the toxicity of plants containing anthraquinones and suggested that these molecules have genotoxic potential due to the presence of the quinone group that either have the capacity to alter the redox system thereby disturbing mitochondrial functions or by nucleophilic addition reactions with biomolecules including DNA and protein.

## 4. Main Anthraquinones with Therapeutic Properties

Among the numerous anthraquinones that nature produces, this paragraph focuses on such compounds with known therapeutic potential and that can have a clear role in diabetes.

### 4.1. Emodin

Emodin (Figure 1(b)) is chemically known as 1,3,8-trihydroxy-6-methylanthraquinone (C_15_H_10_O_5_) and is present in various Chinese medicinal herbs such as active ingredient [4], including *Rheum emodi*, a Himalayan rhubarb [41], *Rheum palmatum* [42], *Polygonum cuspidatum* [43], *Polygonum multiflorum* [44], *Aloe vera* [45], and *Cassia obtusifolia* [46]. These herbs have been used as traditional medicines in many countries, especially in East Asia [32]. This polyphenol has been demonstrated to possess a wide spectrum of pharmacological effects, such as antidiabetic, antiviral, antibacterial, antimicrobial, antiosteoporotic, immunosuppressive, neuroprotective, hepatoprotective, anticancer, anti-inflammatory, antiatherosclerotic, antiallergic, antiulcerogenic, cathartic, diuretic, laxative activities, DNA-binding, and vasorelaxant activities [32, 41, 47–58].

It is reported that emodin is an AMP-activated protein kinase (AMPK) activator with PPAR*γ*-agonist activity [49, 59, 60]. AMPK is a serine/threonine kinase, activated by an increase in the AMP/ATP ratio that regulates the whole body and cellular homeostasis, mitochondrial biogenesis, autophagy, and cell proliferation, and promotes the assembly of adiponectin [49, 61]. Adiponectin is the main adipokine secreted by adipose tissue and is associated with the improvement of insulin resistance and glucose metabolism regulation [62, 63]. Metformin and thiazolidinediones are antidiabetic drugs that indirectly trigger AMPK by inhibition of ATP synthesis and consequently increase AMP levels [64, 65]. It has been reported that AMPK boosts GLUT1/4 levels and mediates glucose uptake [66–69]. Emodin regulates glucose utilization, enhancing GLUT4 translocation and [C-14] glucose uptake by activating AMPK in skeletal muscle and liver cells [69]. Activation of AMPK decreases PPAR*γ* expression [70, 71], a transcription factor that plays an important role in adipocyte differentiation [72]. Emodin activates PPAR*γ* and promotes adiponectin expression and differentiation of 3T3-L1 preadipocytes [49, 73]. Therefore, emodin, as an AMPK activator and a PPAR*γ*-agonist, has both PPAR*γ-*inhibiting and PPAR*γ*-activating activities, so that permits regulating adiponectin expression in opposite ways [49]. This dual activity makes emodin a key modulator and a potential drug candidate for the treatment of type 2 diabetes. Furthermore, emodin has been shown to ameliorate high-fat-diet-induced insulin resistance by reducing lipid accumulation through decreasing fatty acid transport protein 1 (FATP-1) in rat skeletal muscle [74].

Moreover, emodin impacts on inflammation processes. Emodin inhibited the release of TNF*α* from rat basophilic leukemia (RBL-2H3) cells [75]. In LPS stimulated RAW264.7 macrophage cells, emodin suppressed the upregulation of ICAM-1, MCP-1, and TNF*α* and the downregulation of PPAR*γ* [76, 77]. In Wistar rats, emodin reduced corneal inflammation in LPS-induced keratitis due to its capability of inhibition in NF-*κ*B activation [78]. Emodin also exerted protective effects on lung injury in septic rats since a reduction of oxidative stress and inflammation response during sepsis were observed [79]. Furthermore, emodin attenuated cigarette smoke induced lung injury in mouse decreasing the associated inflammation and oxidative damage [80]. Therefore, emodin may affect glucose metabolism due to its anti-inflammatory action and not only through the effect on AMPK [73]. Emodin activated AMPK, downregulated perilipin, and inhibited NF-*κ*B and extracellular signal-regulated kinase (ERK), thereby increasing glycolysis and glucose metabolism and suppressing lipolysis and inflammation [73].

Emodin is the most studied anthraquinone as a potential therapeutic agent against diabetes and the associated complications. However, the data available derive from cell cultures and animal models, whereas clinical trials are still lacking. Most of the animal studies have been performed both in a rat model induced by a high-cholesterol diet plus streptozotocin (STZ) injection that replicates the natural evolution and metabolic characteristics of human type 2 diabetes and in C57BL/6J mice characterized by high susceptibility to diet-induced obesity. In a pioneer study, the effects of emodin were investigated on renal dysfunction in the STZ-induced diabetic rats with nephropathy [30]. Emodin treatment significantly ameliorated the renal dysfunction in diabetic nephropathy rats, reduced serum creatinine and plasma urea nitrogen and proteinuria, but evidenced a weak action on blood glucose levels. Similarly, in another study, the effects of emodin were explored on the podocyte apoptosis in diabetic nephropathy [81]. The expression of phosphorylated protein kinase RNA-like endoplasmic reticulum kinase (P-PERK), phosphorylated eukaryotic initiation factor 2*α* (eIF2*α*), activating transcription factor 4, CCAAT-enhancer-binding protein homologous protein (CHOP), implicated in apoptosis pathway and endoplasmic reticulum (ER) stress response, were decreased. In a recent study, emodin reduced proteinuria and alleviated renal fibrosis without affecting hyperglycemia in STZ-induced diabetic nephropathy rats [82]. The mechanisms involved in emodin renoprotective effects suggested by the authors were the suppression of cell apoptosis and an increase of autophagy of podocytes via the AMPK/mTOR signaling pathway in the kidney.

The expression of phosphorylated p38 mitogen-activated protein kinase (p38 MAPK), cAMP response element-binding protein (CREB), and the downstream target gene fibronectin was downregulated by emodin when compared with the diabetic group. p38 MAPK is a key factor in the MAPK signaling pathway associated with diabetic nephropathy and its inhibition could prevent the development of diabetic nephropathy [83, 84]. Moreover, emodin also reduced proteinuria and fibronectin expression in early-stage of STZ-induced diabetic rats [85]. In another study, emodin administration for two weeks to dyslipidaemic-diabetic STZ rats resulted in a dose-dependent reduction of blood glucose, total cholesterol, triglycerides, free fatty acids, and malondialdehyde and increased plasma superoxide dismutase activity [86]. The authors indicated that the protective effects of emodin were possibly mediated by the upregulation of L-type calcium channels in the pancreas and heart. Similar results in plasma biochemical parameters were also obtained when emodin was tested against a high-fat diet and low dose of STZ-induced diabetic mice [58]. In this study, glucose tolerance and insulin sensitivity were also improved in the emodin group. The results seem to be mediated, at least in part, through the activation of peroxisomal proliferator-activated receptor-*γ* (PPAR*γ*) and the modulation of its downstream metabolism-related genes. In addition to the beneficial effects of emodin on blood glucose levels and lipid profile, this compound also exerted protection against diabetic cardiomyopathy in the same rat model [87]. In this way, type 2 diabetes and cardiovascular complications are closely related to an impaired serine/threonine kinase (Akt)/glycogen synthase kinase 3 beta (GSK-3*β*) pathway. In this study, the animals treated with emodin evidenced an improvement in diabetes-induced systolic dysfunction probably associated with a significant increase in phosphorylation of Akt and GSK-3*β*. The antidiabetic and alpha-glucosidase inhibitory action of diverse 1,8-ihydroxyanthraquinones, like rhein, aloe emodin, emodin, chrysophanol from *Rheum emodi* were evaluated [88]. All anthraquinones tested showed good antihyperglycemic activity, with aloe emodin exhibiting maximum effects. On the contrary, only emodin exhibited potent intestinal alpha-glucosidase inhibition showing a mixed-type inhibition, which could be of great interest in preventing postprandial glucose spikes. The same results were reported more recently, whereas emodin induced the highest *α*-glucosidase and *α*-amylase inhibitory activities in Wistar rats sera [89]. Emodin treatment of fat C57BL/6 mice reduced body weight gain, improved lipid profile, ameliorated insulin sensitivity, and reduced the size of adipocytes [90].

In addition, emodin reduced the mRNA levels of the sterol regulatory element-binding proteins (SREBP), SREBP-1, and SREBP-2, which are involved in the biosynthesis of cholesterol in the liver and adipose tissues, fatty acid, and triglyceride in mammals and also inhibit SREBP transactivity in Huh7 (human hepatoma) cell line [90].

It is known that 11*β*-hydroxysteroid dehydrogenase (11*β*-HSD) 1 enhances local glucocorticoid action by converting cortisone into cortisol in humans, and 11-dehydrocorticosterone into corticosterone in rodents [91]. 11*β*-HSD1 also plays a role in the development of obesity, insulin resistance, and type 2 diabetes [92]. In an *in vitro* assay, emodin showed a potent and selective inhibitory activity against 11*β*-HSD1 [93]. Emodin treatment reversed prednisone-induced insulin resistance in C57BL/6J mice, improved insulin sensitivity and lipid metabolism, lowered blood glucose, and hepatic phosphoenolpyruvate carboxykinase (PEPCK), and glucose-6-phosphatase gene expression. In another study, the inhibition of 11*β*-HSD1 reduced LPS-induced proinflammatory innate immune in 3T3-L1 adipocytes by downregulating phosphatase and tensin homolog (PTEN) expression, leading to activation of the phosphatidylinositol 3-kinase (PI3K)/protein kinase B (PKB) pathway which inhibits inflammation [94, 95]. These effects are in association with an attenuation of the ratio of phosphorylated inhibitor of *κ*B *α* (p-I*κ*B*α*)/I*κ*B*α* and a decrease of NF-*κ*B subunit p50. Emodin treatment of STZ diabetic rats on a high-fat diet also reduced levels of IL-6, PTEN, Cluster of Differentiation 68 (CD68), and the ratio of p-I*κβα* to I*κβα* in visceral fat [95].

Different studies investigated the *in vitro* effects of emodin in multiple cell models. In a first approach, the effects of emodin on adiponectin expression and multimerization were investigated in 3T3-L1 adipocytes and in human embryonic kidney 293T cells. The results evidenced that emodin activated AMPK in both cell types and promoted the assembly of adiponectin in 3T3-L1 adipocytes. In addition, emodin activated PPAR*γ,* thus promoting the differentiation of preadipocytes and the expression of adiponectin. The authors suggested that observed effects on adiponectin were the final effects resulting from both AMPK activation and PPAR*γ* activation. Another study investigated the capability of emodin-6-O-*β*-D-glucoside to protect against the vascular inflammatory process, which is directly associated with various diabetic complications induced by high glucose in primary human umbilical vein endothelial cells (HUVECs) and in mice [96]. The acute treatment with emodin-6-O-*β*-D-glucoside significantly reduced vascular permeability, monocyte adhesion, expression of cell adhesion molecules (VCAM-1, ICAM-1, and E-selectin), formation of ROS, and activation of NF-*κ*B, which were induced by high glucose concentration. Using the same endothelial cell model, the beneficial effects of emodin were evaluated in cytotoxicity tests induced by high glucose. HUVECs cells evidenced significant damage, which was prevented coculture with emodin [97]. The protective effects of emodin might be related to the inhibition of Chemokine C-C motif ligand 5 (CCL5) expression and reduced adhesion of monocytes to HUVECs. Emodin also suppressed activation of p38 MAPK and ERK1/2 due to high glucose levels. The same research group reported that hyperglycemia could induce proliferation and decreased apoptosis of mesangial cells leading to renal dysfunction by upregulating cellular FLICE-inhibitory protein (cFLIP) [85]. Emodin treatment normalized this alteration via inhibiting cFLIP in rat glomerular mesangial cells C line (HBZY-1) which, in turn, promoted apoptosis and repressed proliferation. Aldose reductase is a member of the aldo-keto reductase superfamily that catalyzes the reduction of glucose to its sugar alcohol sorbitol, playing an important role in the pathogenesis of diabetic cataracts [98]. Emodin has been reported to exert good selective inhibitory activity against aldose reductase both in *in vitro* and in transgenic mice suggesting a potential therapeutic in the prevention of cataracts in diabetic patients [99]. Furthermore, emodin can also impact diabetic retinopathy and cataract through inhibition of retinal neovascularization via modulation of HIF-1*α*/VEGF signaling pathway and through decreasing expressions of VEGFA, HIF-1*α,* and PHD-2 [81].

Another point to consider is the potential therapeutic effects of emodin in autoimmune diabetes (type I diabetes) associated with its anti-inflammatory effects. Inflammation is involved in insulitis and *β*-cell destruction in type I diabetes [19]. It has been reported the emodin could suppress the C-X-C chemokine receptor type 4 (CXCR4) chemotactic activity of leukocytes towards pancreatic islets at the insulitis stage of autoimmune diabetes development [100,101]. This suppression has been evidenced both in *in vitro* and in animal models and involved emodin-mediated inhibition of MAPK pathways. In addition, emodin could enhance HIF-1*α* and GSK-3*β* levels in rats with severe acute pancreatitis (SAP), suggesting its protective role in such conditions [102]. The same experimental model (SAP) was used to describe the decrease of serum amylase and related inflammatory cytokines (TNF-*α* and IL-6) thanks to the inhibition of JNK and p38 MAPK phosphorylation in the animal pancreas [103]. The authors reported that such biomolecular effects of emodin were partially due to the blockade of ER stress transducers IRE1*α* and its downstream molecules. Moreover, emodin could induce apoptosis of inflammation-related lymphocytes and could diminish the expression of pre-B-cell colony-enhancing factor (PBEF) in rat peripheral blood PMNs. PBEF is a key factor for inflammation and oxidative stress, influencing neutrophil infiltration and alveolar permeability [103].

### 4.2. Diacerein

Diacerein (Figure 1(c)) is another naturally occurring anthraquinone with the chemical formula 1,8-diacetoxy-3-carboxylanthraquinone (C_19_H_12_O_8_). This molecule is a symptomatic slow-acting drug in osteoarthritis, and its active metabolite is rhein (1,8-dihydroxy-3-carboxylanthraquinone, C_15_H_8_O_6_, Figure 1(d)) [19, 104]. As diacerein shows poor aqueous solubility and partial bioavailability, recently, a tentative to improve its pharmacological properties has been reported [105]. A specific preparation of solid dispersion systems for enhanced dissolution of diacerein has been tested in healthy adults and geriatrics, reporting bioavailability enhancement of the optimized solid dispersion of diacerein. Similarly, other studies have pursued the same aim, the improvement of diacerein solubility and bioavailability [106–110].

As diacerein is well known for its antiosteoarthritis effects, numerous works analyzed its functions in preclinical and clinical settings. For a comprehensive review on such effects, the reader can refer to two recent works that analyzed extensively the antiosteoarthritis properties of diacerein [111, 112]. Nonetheless, a very recent clinical trial compared diacerein to celecoxib in patients affected by knee osteoarthritis [113]. The study reported that more than 350 subjects were randomly treated with diacerein and celecoxib for 6 months. Measured outcomes showed no significant difference between treatment groups, with diacerein being statistically comparable to celecoxib in knee osteoarthritis treatment. The polyphenol had a good safety profile, even if a rare adverse effect was diarrhea (10.2% vs. 3.7% for celecoxib).

The principal mechanism of action of diacerein is to decrease inflammatory cytokines via inhibition of interleukin-1*β* (IL-1*β*) system and related downstream signaling [114]. Moreover, diacerein has revealed other properties such anticatabolic and proanabolic effects on cartilage and synovial membrane [115]. Diacerein induced the activation of IL-1*β* via reduced production of IL-1 converting enzyme [116], in addition to affecting the sensibility to IL-1 by decreasing IL-1 receptor [117], and by indirectly increasing IL-1 receptor antagonist production [118, 119]. Therefore, diacerein decreased cytokine concentrations, in particular IL-1*β* and TNF*α* [120–122], both of them involved in pancreatic *β*-cell apoptosis and in failure of insulin secretion [122–124]. Different studies have related an improvement in pancreatic *β*-cell function and insulin secretion with several pharmacological interventions such as anakinra [125], etanercept [126], nonsteroidal anti-inflammatory drugs [127], or thiazolidinediones [128]. As a result, diacerein has potential usefulness for the treatment of type 2 diabetes, given the inhibiting effects on IL-1*β* and TNF*α* [122] and the improvement of the hepatic glucose metabolism [129]. Consequently, diacerein has been successfully used to treat autoimmune diabetes in nonobese diabetic mice similar to its active metabolite rhein [100].

Diacerein has been showed to alleviate pain in patients with osteoarthritis and joint pain. Moreover, it has been studied in *in vitro* and *in vivo* experimental models. For example, it exhibited a high inhibitory activity on N-acylethanolamine-hydrolyzing acid amidase (NAAA), a compound implicated in pronociceptive effects through inhibition of palmitoylethanolamide (PEA), demonstrating an analgesic effect [130]. The polyphenol was able to block glutamatergic transmission through both ionotropic and metabotropic receptors (antinociceptive effect), together with a reduction of IL-1*β* and TNF-*α* [131]. Further, diacerein showed antiedematogenic and chondroprotective effects (in addition to antinociceptive), reducing metalloproteinase (MMP)‐9 and transient receptor potential vanilloid 1 (TRPV1) expression in the spinal cord of rats, in addition to astroglial inhibition [132].

Diacerein has been studied in diabetes preclinical models. The first study related to diabetes and diacerein was performed in nonobese diabetic mice (high similarity with human type 1 diabetes mellitus) [121]. The suffered damage of the *β*-cells has been related to augmented levels of proinflammatory cytokines [133]. In that work, several doses were used to test diacerein (5, 10, and 50 mg/kg/day i.p. during 24 weeks, *n* = 30/group) in serum samples. A 40% reduction in the incidence of diabetes was observed after the treatment with the medium dose (10 mg/kg/day). Also, a reduction of the proinflammatory cytokines (IL-1*β*, IL-12, TNF-*α*) in serum was observed in diabetic animals when compared with the control group. However, an increase in cytokine genes expression in pancreatic cells was observed in nondiabetic mice (no data is available for diabetic animals). Consequently, the authors suggested that diacerein might induce a posttranscriptional or a posttranslational downregulation of cytokines in these animals. In another study, mice fed with a high-fat diet were used as an animal model of type 2 diabetes mellitus in which insulin resistance was the main metabolic disturbance [129, 134]. TNF- *α*, IL-6, and IL-1*β* mRNA expressions in several tissues (liver, muscle, and adipose tissue) and inflammatory cytokines (IL-6 and TNF-*α*) in serum were diminished in animals who received the high-fat diet and diacerein during 10 days (20 mg/kg/day) when compared with the ones that did not receive diacerein and reaching values similar to controls (animal with a standard diet). They described an improvement in glucose tolerance and a decrease in macrophage infiltration in adipocytes. In addition, similar results were found when authors studied the phosphorylation status of PKR-like ER kinase (PERK) [134], an indicator of ER stress since it was previously related to the inflammation process [135].

While different clinical trials have been performed until now on diacerein and diabetes, our review only discusses two very recent meta-analyses that deeply analyzed the effects of diacerein in humans. The first meta-analysis proposed by Guo and collaborators studied diacerein supplementation in T2DM patients in randomized controlled trials (RCTs). FBG, HbA1c, body mass index (BMI), C-reactive protein (CRP) were significantly improved in the treatment group. However, the number of works included (5) and the number of subjects (278) were relatively small, and the authors suggested the more consistent population studies were necessary to better validate such data [136]. Another meta-analysis reported different results for FBG that was not modified by diacerein treatment [137]. However, HbA1c and BMI were ameliorated as in the previous meta-analysis. These apparently conflicting results can be imputable to the different works analyzed and thus to the number of patients. Nonetheless, both the works came to the same conclusions: diacerein could provide a substantial contribution to T2DM, with better glycaemic control and a reduced body weight. The supplementation of this polyphenol could represent an effective therapeutic tool if wide RCTs will confirm these data.

In relation to glycaemic control, diacerein can impact 3 target organs: adipose tissue, liver, and skeletal muscle. In the adipose tissue, the polyphenol reduces ER stress, proinflammatory cytokines, protein-tyrosine phosphatase 1B (PTP1B) while increasing phosphorylated Akt and insulin receptor substrate 1 (IRS-1). In the liver, a drop of inflammatory status and PTP1B was reported, with augmentation of glucose uptake. In the skeletal muscle, inflammatory status, ER stress, PTP1B, gluconeogenesis, fasting plasma glucose, and fatty acid oxidation were decremented, while phosphorylated Akt and IRS-1 were amplified.

### 4.3. Catenarin

Another interesting naturally occurring anthraquinone derivative is catenarin (Figure 1(e)) with chemical formula 1,4,6,8-tetrahydroxy-3-methylanthraquinone (C_15_H_10_O_6_). Catenarin presents higher antichemotactic activity than other anthraquinones for the number and position of hydroxyl groups in the anthraquinone structure [19, 100]. This compound prevents type 1 diabetes in nonobese diabetic mice via inhibition of leukocyte migration mediated by CCR5 and CXCR4 via the inactivation of MAPKs (p38 and JNK), MKKs (MKK6 and MKK7), and calcium mobilization [19, 100]. At the autoimmune diabetes onset in patients and animal models, leukocytes infiltrate into the pancreatic islets, a condition termed insulitis [47, 138]. This invasion is mediated mainly by *T* and B lymphocytes, dendritic cells, macrophages, and natural killers cells and contributes to a gradual loss of pancreatic *β*-cells, thus leading to an insulin deficiency or insufficiency and afterward hyperglycemia [139]. Catenarin and derivatives can suppress diabetes via inhibition of leukocyte infiltration and consequently insulitis [19, 100]. Cascarin (emodin-6-O-rhamnoside, C_21_H_20_O_9_, Figure 1(g)) and physcion (emodin 3-methyl eter, C_16_H_12_O_5_, Figure 1(f)) are other anthraquinones which suppress the chemotactic activity of leukocytes at the insulitis stage during autoimmune diabetes development [100]. Emodin and physcion display kinase and tyrosinase inhibition activity [140, 141], also showing anticancer properties [52, 142].

The position of the hydroxyl group seems fundamental for the antichemotactic activity of anthraquinones. Catenarin has four hydroxyl groups, two of them at R4 and R6 in its anthraquinone ring; emodin has three hydroxyl groups, one of them at R6; cascarin, rhein, and physcion have two hydroxyl groups but any of them at R4 and R6; and diacerein has no hydroxyl group. Of these compounds, catenarin has the highest antichemotactic activity, followed by emodin, cascarin, and rhein [100, 101]. The presence of the hydroxyl at R4 and R6 in anthraquinones seems to be related to antichemotactic activity [19, 100, 101].

Up to now, only one study has been published focusing on the mechanisms and pathways in which catenarin acts in a mice diabetes model [100]. The authors observed that this anthraquinone (4, 20, and 40 mg/kg i.p., 3 times/week, during 26 weeks) prevented type 1 diabetes in nonobese diabetic mice in a dose-dependent manner. The authors reported a reduction of blood glucose and HbA1c levels after the 26 weeks of treatment when compared to the control group. Likewise, leukocyte migration typical in this type of diabetes was suppressed by catenarin via inhibition of the chemokine receptors CXCR4 and CCR5 in T cells.

## 5. Conclusions and Future Perspectives

Diabetes is a complex condition in which high glucose levels occur since the body does not use it properly or is incorrectly synthesized. Several treatments have been attempted in the last decades, but natural products could reserve a wide source of novel molecules to counteract this disease. According to the last piece of evidences on anthraquinones, emodin seems the most prosing since its anti-inflammatory and antioxidant activities could ameliorate or reduce the diabetic symptoms and control the related cytokines pathways. Although the synthesis of new emodin derivatives has been increasing in the last years, a lack of preclinical studies testing these derivatives related to the diabetic condition is still scarce. In conclusion, the antidiabetic effects of emodin, diacerein, and catenarin need to be rapidly and thoroughly studied in future clinical trials to assess their benefits on diabetic patients.

## Data Availability

No data were used to support this study.
